# A Needle-Free Injection System (INJEX™) with lidocaine for epidural needle insertion: A randomized controlled trial

**DOI:** 10.12669/pjms.323.9174

**Published:** 2016

**Authors:** Muhammet Gozdemir, Ruveyda Irem Demircioglu, Safinaz Karabayirli, Huseyin Sert, Bunyamin Muslu, Burhanettin Usta, Ummugulsum Yazici

**Affiliations:** 1 Muhammet Gozdemir, MD. Associate Professor, Anesthesiologist, Department of Anesthesiology, Faculty of Medicine, Turgut Ozal University, Ankara, Turkey; 2 Ruveyda Irem Demircioglu, MD. Associate Professor, Anesthesiologist, Department of Anesthesiology, Faculty of Medicine, Turgut Ozal University, Ankara, Turkey; 3 Safinaz Karabayirli, MD. Assistant Professor, Anesthesiologist, Department of Anesthesiology, Faculty of Medicine, Turgut Ozal University, Ankara, Turkey; 4 Huseyin Sert, MD. Associate Professor, Anesthesiologist, Department of Anesthesiology, Faculty of Medicine, Turgut Ozal University, Ankara, Turkey; 5 Bunyamin Muslu, MD. Associate Professor, Anesthesiologist, Department of Anesthesiology, Faculty of Medicine, Turgut Ozal University, Ankara, Turkey; 6 Burhanettin Usta, MD. Associate Professor, Anesthesiologist, Department of Anesthesiology, Faculty of Medicine, Turgut Ozal University, Ankara, Turkey; 7 Dr. Ummugulsum Yazici, MD. Anesthesiologist, Department of Anesthesiology, Faculty of Medicine, Turgut Ozal University, Ankara, Turkey

**Keywords:** Needle Free, Injection, Local Anesthetic delivery, Technology

## Abstract

**Objectives:**

Local anesthetic infiltration is also a process of a painful process itself. INJEX™ technology, known as “Needle-free” drug delivery system, was designed for reducing the pain associated with cutaneous procedures. We conducted a prospective, randomized trial to evaluate the application of lidocaine with INJEX™ system and 27-gauge needle.

**Methods:**

A total of 60 consecutive patients were allocated to receive either INJEX group or 27-gauge needle group. Local anesthetic infiltration was applied two minutes before epidural needle insertion.

**Results:**

Mean VAS, at the time of local anesthetic injection was 0 for group I and 2 for group II. When the effect of epidural needle insertion was compared, the mean VAS score was one versus two for Group-I versus Group-II, respectively. Lidocaine applied with the INJEX™ system before epidural needle insertion significantly reduced the intensity of pain during that procedure and was least effective the lidocaine applied with the 27-gauge needle and patients felt less pain during at the time of local anesthetic injection in Group-I.

**Conclusion:**

Needle-free delivery of lidocaine is an effective, easy to-use and noninvasive method of providing local anesthesia for the epidural needle insertion.

## INTRODUCTION

Regional anesthesia for caesarean section is a common procedure. Especially for epidural anesthesia, local anesthesia before the epidural needle insertion routinely required. But local anesthetic infiltration is also painful process itself. Different methods have been proposed to alleviate the pain associated with epidural needle insertion. Local anesthetic infiltration of lidocaine with a 25-30-gauge needle, lidocaine patch and eutectic mixture of local anesthetics (EMLA) cream are the most widely used methods.

Most of the commercially available topical anesthetics require a relatively long application time to reach satisfactory analgesia, ranging from 30 to 90 min.^1^,^2^ This may be justified in busy day-medicine units, where application of EMLA^®^ and lidocaine patch delay regional anesthesia. Local anesthetic infiltration may also a painful method^3^ and fear of needle is a very common reason for patients refusing regional anesthesia. Needle phobia affects at least 10% of general population.^4^ Therefore a painless, effective and rapid method is required. INJEX™ technology, a system, known as “Needle-Free” drug delivery system, was designed for reducing the pain associated with cutaneous procedures that involve needle insertion such as venipuncture, medications and local anesthetics.^5^-^12^

Needle free injection systems are advanced systems that work by forcing the liquid medication at an elevated speed through a small orifice without piercing the skin. In this system high pressure allows the more rapidly administration of drugs by penetrating to the skin in devoid of an injection. Since the high-pressure fluid stream powers its way through the tissue, it results in a wider distribution of the medication especially in the least resistant tissues. Also the avoidance of needle stick accidents is another advantage of this system that has both reusable and disposable forms. These systems work mechanism is that it is held against the skin. Due to this an ultrafine stream of fluid is created, that penetrates the skin devoid of the use of a needle. INJEX™ technology improves the dispersion of medication throughout the tissue as compared to needle-based injection. This creates an ultra-fine stream of high-pressure fluid that penetrates the skin without using a needle. Needle-free injection relies on a high-pressure stream of the medication itself to penetrate the skin. As the fluid stream forces its way through the tissue, it follows the path of least resistance, resulting in a widely dispersed, spider web-like distribution of the medication. Moreover, they should decrease the occurrence of needle stick injuries that have been seen in some health care workers contracting diseases. Devices are available in reusable and disposable forms and also in versions for multiple patients and institutional uses. Production of needle free devices is totally controlled by FDA due to the safety issues.

This system has been found effective and reliable, and has been shown to cause less pain for patients with its widely usage.^7^,^10^ We therefore conducted a prospective, randomized trial to evaluate the efficacy and safety of INJEX™ system with lidocaine for the insertion of epidural needle.

## METHODS

After institutional review board approval, a prospective randomized study was performed in ASA I -II, aged 18-45 years in 60 women at term pregnancy (≥ 36 weeks) scheduled for elective caesarean section delivery. Exclusion criteria included, known allergic reaction to lidocaine, contraindications to regional anesthesia, the refusal of the patient’s regional anesthesia and intellectual disabilities that prevented completion of a visual analog scale (VAS). All patients were instructed for using 10 cm VAS during the preoperative period. Randomization via computer processing was performed using random numbers.

All received 10 mL/kg of warmed crystalloid intravenously over a period of 15 minutes before regional anesthesia. Monitoring included ECG, pulse oximetry and noninvasive blood pressure measurement cycled at three minutes intervals (Datex Engstrom AS/3 Anesthesia Monitor; Helsinki, Finland). All patients received combined spinal epidural (CSE) anesthesia. The blocks were performed at L_3-4_ or L_4-5_ interspaces with the parturient’s in the sitting position.

**Fig.1:**
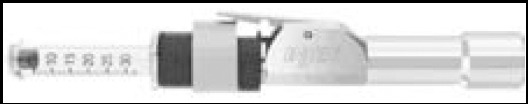
Needle-free injection system; INJEX™ model 210.

Injections to Group 1 patients (Group I) with 27-gauge needle (Set syringe, Turkey) and 1.5 ml 2% lidocaine hydrochloride (Jetmonal 2% ampoule, ADEKA, Turkey) was performed with the skin and subcutaneous tissue infiltration. Group 2 patients were administered (Group II) with 0.3 ml 10% lidocaine hydrochloride through needle-free injection system INJEX™ model 210 (Rösch, Berlin, Germany) which was applied with the skin and subcutaneous tissue infiltration. (Fig.1). Skin was disinfected by povidon iodine before performing INJEX and then again disinfected by the same way. After that the gloves were changed and the region was covered with surgical drapes. Patients’ pain scores during the application of local anesthetic were recorded. Two minutes after local anesthesia, 18-gauge Tuohy needle (Espocan; B.Braun, Melsungen, Germany) was introduced using loss of resistance to air, and the duramater was punctured with a 27-gauge Quincke needle using the “needle-through-needle” technique. Patients’ pain scores during the epidural needle insertion and each patient’s number of attempts for epidural needle insertion were recorded. Pain intensity was assessed at the time of local anesthetic injection and during epidural needle insertion by 10 cm VAS scale. Patient satisfaction in epidural anesthesia were assessed on a four-point scale (0 = poor, 1 = medium, 2 = good, 3 = excellent) by the same process and, they were asked if they would prefer the same for their next procedure. Local skin reactions at the injection site (erythema, edema, hemorrhage, etc.) were also recorded.

The needle free injection systems comprises of three components; nozzle, drug reservoir and pressure source. The nozzle has a flat surface and an orifice that contacts with the skin and provides the passage of the drug. The orifice of the nozzle is the main mechanism controlling the drug stream speed. The drug reservoir is a syringe that touches the skin and should be sterilized or disposed prior to the administration. The pressure source is the energy source required for the drug administration. The stored energy is released by pushing a plunger to deliver the required pressure. When administered through the skin, an ultrafine stream of fluid penetrates the skin and provides the administration of the drug in a time period shorter than a second.

Statistical analyses were performed using SPSS 13.0 version (SPSS, inc. Chicago, IL, USA). Power analysis was performed for the preliminary study with 10 patients. The VAS score while inserting the epidural needle was 0.7 for Group I and 2.1 for Group II with common standart deviation of 2.1. Each group was calculated consisting of at least 28 patients with,α value 0.5, β value 0.80.

Normal distribution of data availability was assessed by the Shapiro-Wilk test and histogram. Demographic data with the Student’s t-test, VAS values during application of local anesthesia and epidural needle insertion with the Mann-Whitney U-test were used. Comparison of complications, applied interval, epidural needle number of attempts, whether the same process was preferred again and patient satisfaction were analyzed with the chi-square test. Numeric values were expressed as median (25%-75%) and mean±SD, p values less than 0.05 were considered to be significant.

## RESULTS

A total of 60 consecutive patients were divided into group I (n=30) or group II (n=30). The patients assigned to each group were comparable with respect to demographic data, applied interval and number of attempts of epidural needle and there were not significant differences between the two groups (Table-I).

**Table-I:** Demographic Data.

	*Group I (n=30)*	*Group II (n=30)*	*P-value*
Age (yr)	31±5	30±4	0.311
Height (cm)	164±5	167±6	0.17
Weight (kg)	78±9	76±9	0.376
Applied interval (L_3-4_/L_4-5_)	18/12	23/7	0.165
*Number of attempts of ENI, n (%)*			
1	26	27	0.431
2	4	2	
>2	0	1	

Values are means±SD and number of patients.

ENI: Epidural needle insertion, No significant differences were observed.

**Fig.2:**
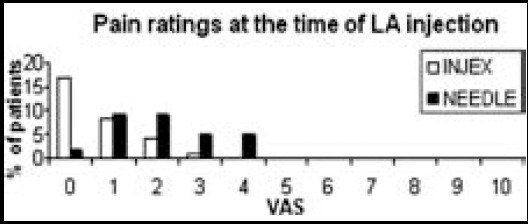
Pain ratings at the time of local anesthetic injection.

There was a significant difference (p=0.001) in pain ratings at the time of local anesthetic injection between group I [0 (0-3)]; and group II [2 (0-4)]. But, there was no difference (p=0.074) in VAS during epidural needle insertion between the group I [1 (0-4)] and the group II [2 (0-5)] (Table-II) (Fig.2).

**Table-II:** Patient’s VAS values and local injection site reaction.

	*Group I (n=30)*	*Group II (n=30)*	*p-value*
LA VAS values when implementing	0 [0-3]*	2 [0-4]	0.001
EN VAS values when implementing	1 [0-4]	2 [0-5]	0.074
*Local injection site reaction*			
Bleeding	9	7	0.559
Erythema	6	3	0.217
Edema	5*	0	0.001

Values are median (range) (%25-%75) and number of patients. LA: Local Anesthesi, EN: Epidural Needle, VAS: Visual Analogue Scale

* p<0 05 compared to the two groups.

**Fig.3:**
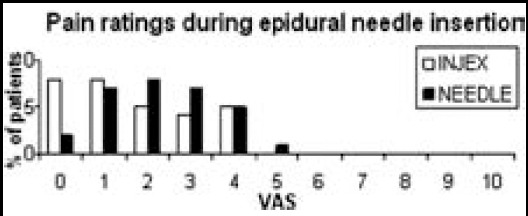
Pain ratings during epidural needle insertion.

Patients satisfaction rates were as follows: 2 vs 0 patients rated excellent, 18 vs 12 patients rated good, 10 vs 11 patients rated poor and 0 vs 3 patients rated fair in group I compared to Group II (p=0.160). Although the patient satisfaction was slightly higher in group I, the difference between the groups was not statistically significant. The number of patients who chose the same method for local anesthesia were 28 vs 25 (p=0.424) in group I compared to group II (Table-III) (Fig.3).

**Table-III:** Patient Satisfaction.

	*Group I (n=30)*	*Group II (n=30)*
Excellent	2	0
Good	18	16
Fair	10	11
Poor	0	3
Preferred rate again (Yes/No)	28/2	25/5

Values are number of patients. No significant differences were observed.

29.7% vs 23.1% had very slightly erythema of the skin (p=0.559), 19.8% vs 9.9% had minor local bleeding (p=0.217) in group I vs group II, respectively. Edema was more common in the group I than group II (16.5% vs 0%, p=0.001) (Table-III). Treatment-related adverse events were generally mild and self-limiting.

## DISCUSSION

We found that patients felt less pain during the application of local anesthetic with the INJEX™ system than 27-gauge needle. But, the pain of inserting the epidural needle was similar in both groups.

Various methods have been developed to alleviate pain before epidural needle insertion. Many researchers have used lidocaine tape and EMLA^®^ cream for epidural and spinal anesthesia, which provide adequate analgesia, although less effective than lidocaine infiltration during the administration of spinal or epidural anesthesia. Main disadvantage of this method is that it should be applied at least 30-90 minutes before the procedure to achieve topical anesthesia and side effects related prilocaine can be seen.^1^,^2^,^15^,^16^ Elson et al. determined the efficacy of EMLA and lidocaine infiltration for 16-gauge Tuohy needle insertion. They concluded that, the application of EMLA cream for at least 90 minutes plus 1% lignocaine infiltration optimized patient comfort for epidural insertion.^14^

The most popular method employed in patients for reduction of injection pain is full infiltration with a 25-30-gauge needle using lidocaine 1-2%.^17^ Although this method is very effective, its application is difficult for patients with needle phobia. Needle phobia represents a serious problem for at least 10% of the population.^4^ Refusal of regional anesthesia by obstetric patients was investigated previously by Gajraj et al., and it was found that the most common reason for refusal was fear of needle placement rate of 28%.^18^ For this reason a noninvasive, nontraumatic and painless method that provides a rapid onset of analgesia is required. INJEX™ technology, known as “Needle-free” drug delivery system, is based on the principle of using a mechanical energy source to create a release of pressure sufficient to push a dose of liquid medication through a very small orifice. It is a reusable system that uses disposable ampoules, without the use of a needle. After skin contact, less than 0.3 ml can be injected with a 3000 psi pressure to the skin-subcutaneous tissue (5-8 mm depth) in 0.2 seconds. INJEX™ is an alternative to needle syringes and is noninvasive, nontraumatic, safe, effective and almost painless. The analgesic effect occurs within 2-3 minutes after administration.^6^,^19^,^20^

Although fear of injection-related pain is widespread among patients, the concept of needle-free injection is only known to a limited number of people. Efficacy of the needle-free injection of local anesthesia for reducing the pain associated with other cutaneous procedures that involve intravenous catheter placement, digital ring block, fine-needle aspiration biopsy or spinal needle insertion was previously reported and it was shown that it significantly reduced patients’ perceived pain intensity.^5^-^10^,^21^ Cooper et al., compared needleless injector (J-Tip) and a 25-gauge needle for large-bore intravenous cannula insertion. A subcutaneous injection of 0.3 ml of 1% plain lidocaine was administered three minutes prior to the procedure. They found that, the injection with the needleless injector caused significantly less pain than with 25-gauge needle, however, more pain was observed on cannulation.^6^

Lysakowski et al., evaluated lidocaine dose-response, of the J-tip injector for pain relief associated with the insertion of 18-gauge cannula into dorsum of the hand and they found that, a total dose of 0.5 ml of 2% lidocaine produced significantly greater analgesia compared to 1% lidocaine.^20^ In the study by Jimenez et al, the efficacy of J-Tip and EMLA in reducing pain during IV cannulation and placement was compared. Patients received 1% buffered lidocaine via J-Tip (0.25 mg) or EMLA (2.5 g) prior to cannulation. Pain was rated via visual analogue scale scores (1-10) at the time of venous cannulation. Median pain score was 0 in the J-type group and three in the EMLA group.^22^

Needleless instruments are well accepted among both adult and pediatric populations; however their effects to prevent the pain of epidural needle insertion have not been studied. In our study, lidocaine injected via INJEX™ was more effective than injection via a 27-gauge needle during the epidural needle insertion. Pain intensity was assessed twice: at the time of local anesthetic injection and during the epidural needle insertion. VAS scores at the time of lidocaine infiltration (0 vs 2, p=0.001) were significantly less Group I than Group II, but VAS scores during the epidural needle insertion were not significantly different between the two groups (1 vs 2, p=0.074). The INJEX group had slightly less pain during the epidural needle insertion but this difference is statistically insignificant. This might be caused by spreading of the drug to a larger area of tissue when high-pressure is used.^24^ Patients in the INJEX group experienced significantly less pain at the time of lidocaine injection. In a prior report, the application of lidocaine with INJEX™ was shown to be an effective alternative to 27-gauge needle for lidocaine infiltration since it caused less pain and more patient satisfaction.^21^

Increases in erythema and minor bleeding were seen after treatment with INJEX™, however, these dermal reactions were minor, short-lived, and self-limited. Lysakowski et al. evaluated the J-tip injector for the dorsum of the hand and observed that it produced transient local hyperemia in 13% and minor bleeding 17% of the patients. Migdal at al reported that powder jet that they use in pediatric patients was associated with a small increase in erythema and petechial at the injection site.^19^,^20^ In our study, dermal reactions, especially edema with slight raising on edges of the area at the injection site was observed more frequently in group I than group II. This may be due to the fact that all of the patients were pregnant and the high-pressure injection of the local anesthetic caused subsequent local edema and minor bleeding. In a prior report, dermal reactions were a little more frequent in INJEX group than 27-gauge needle group, but no cases of edema developed.^21^

Patient satisfaction was a little bit higher in group I (p=0.424). The total numbers of patients with excellent and good scores were 20 vs 16 patients in group I vs in group II. Three patients found that the method was poor in dental group. Two patients in group I and 5 patients in group II did not choose local anesthesia practice again.

The advantages of using the needleless systems for delivery of local anesthetics are easy, with higher patient acceptance and intences of needle fobia. Main disavantages are higher cost, the potential to frighten the patients due to the sudden noise produced, intense pressure sensations that occur upon the delivery of the anesthetic and the intrusive appearance and need for personnel training and maintenance. Needle-free drug delivery system is costly but reduces cost of disposal, obviates needle-stick injury and eliminates the risks of transmitting infectious diseases like Hepatitis and HIV by contaminated needle stick accidents.

INJEX™ technology could have positive outcomes in epidural anesthesia for patients. To our knowledge, this is the first report of the effectiveness of a needle-free system in management of pain associated with epidural needle insertion. Needle-free system is a rapid, easy to-use method and causes less pain than that produced by a needle-stick. Considering the success of this technique, this method can be applied especially to patients with needle phobia.

The reuse of needles may cause the transmission of many diseases that can be prevented by the use of Needle free injection technology. Moreover, very rapid injection and wide distribution of the drugs are the other benefits of this system. We concluded that INJEX^®^ of local anesthetic should be considered prior to during epidural needle insertion.
